# Identification of Genes Implicated in Methapyrilene-Induced Hepatotoxicity by Comparing Differential Gene Expression in Target and Nontarget Tissue

**DOI:** 10.1289/ehp.9396

**Published:** 2007-01-17

**Authors:** J. Todd Auman, Jeff Chou, Kevin Gerrish, Qihong Huang, Supriya Jayadev, Kerry Blanchard, Richard S. Paules

**Affiliations:** 1 National Institute of Environmental Health Sciences, National Institutes of Health, Department of Health and Human Services, Research Triangle Park, North Carolina, USA; 2 Boehringer Ingelheim Pharmaceuticals, Inc., Ridgefield, Connecticut, USA

**Keywords:** DNA microarray, gene expression, hepatotoxicity, liver, methapyrilene, toxicogenomics

## Abstract

**Background:**

Toxicogenomics experiments often reveal thousands of transcript alterations that are related to multiple processes, making it difficult to identify key gene changes that are related to the toxicity of interest.

**Objectives:**

The objective of this study was to compare gene expression changes in a nontarget tissue to the target tissue for toxicity to help identify toxicity-related genes.

**Methods:**

Male rats were given the hepatotoxicant methapyrilene at two dose levels, with livers and kidneys removed 24 hr after one, three, and seven doses for gene expression analysis. To identify gene changes likely to be related to toxicity, we analyzed genes on the basis of their temporal pattern of change using a program developed at the National Institute of Environmental Health Sciences, termed “EPIG” (extracting gene expression patterns and identifying co-expressed genes).

**Results:**

High-dose methapyrilene elicited hepatic damage that increased in severity with the number of doses, whereas no treatment-related lesions were observed in the kidney. High-dose methapyrilene elicited thousands of gene changes in the liver at each time point, whereas many fewer gene changes were observed in the kidney. EPIG analysis identified patterns of gene expression correlated to the observed toxicity, including genes associated with endoplasmic reticulum stress and the unfolded protein response.

**Conclusions:**

By factoring in dose level, number of doses, and tissue into the analysis of gene expression elicited by methapyrilene, we were able to identify genes likely to not be implicated in toxicity, thereby allowing us to focus on a subset of genes to identify toxicity-related processes.

The advent of DNA microarray technology has spurred the recent growth of toxicogenomics studies [reviewed in Waters and Fostel (2004)]. By measuring changes in gene expression after toxicant exposure on a genomewide scale, investigators can attempt to identify genes or pathways involved in the mechanism of toxicity for that particular toxicant. However, because of the nature of global gene expression profiling, many of the genes found to be differentially expressed may not be related to toxicity. For example, some genes may change because of altered feeding schedules or diurnal rhythms (Boorman et al. 2005; Kita et al. 2002), whereas other gene changes may be related to the pharmacology but not toxicology of the administered substance. Careful design of toxicogenomics studies can reduce the complexities of analyzing gene expression data, such as using time-matched controls to remove those genes for which expression values change with diurnal rhythms. In addition, using different doses in toxicogenomics studies, ranging from pharmacologic/nontoxic to minimally toxic to highly toxic, can often identify genes that are responding to the pharmacologic properties of the administered toxicant.

Methapyrilene, an antihistaminic compound removed from the U.S. market after it was found to lead to the development of hepatic cancers in rats (Lijinsky et al. 1980), has been the focus of several toxicogenomics studies (Beekman et al. 2006; Hamadeh et al. 2002; Waring et al. 2004). The Hamadeh et al. and Waring et al. studies examined hepatic gene expression in rats treated from 1 to 7 days with methapyrilene at doses of 10 mg/kg and 100 mg/kg, whereas the Beekman et al. study examined expression changes in hepatocytes exposed *in vitro* to methapyrilene. The Hamadeh et al. study examined hepatic gene expression to try to correlate gene expression changes with alterations in histopathology after methapyrilene treatment to identify genes involved in methapyrilene-mediated hepatotoxicity (Hamadeh et al. 2002). In the Waring et al. study, the authors demonstrated the robustness of toxicogenomics techniques by showing that several different sites produced concordant results after performing gene expression analysis (Waring et al. 2004). Although using different microarray platforms, both studies produced similar results in gene expression changes after methapyrilene treatment. However, because the low dose of methapyrilene administered for 7 days resulted in the appearance of hepatotoxicity (Hamadeh et al. 2002), it was difficult to identify gene changes to be excluded that are due to the pharmacologic effect of methapyrilene administration. Although many of the gene changes induced by methapyrilene are involved in the mechanism of methapyrilene-mediated hepatotoxicity, it is quite likely that some of the genes and pathways identified are not related to the development of toxicity.

Thus, to facilitate the identification of toxicity-related genes, we compared gene changes in a nontarget tissue for toxicity—the kidney—with those of the target organ of methapyrilene toxicity—the liver—in addition to analyzing gene expression changes across dose level and number of doses. To accomplish this goal, we analyzed global gene expression changes using microarrays from RNA isolated from kidneys obtained from the Hamadeh et al. (2002) study, in addition to reanalyzing the liver RNA generated from this same study. The combination of all three factors in this experiment (dose level, number of doses, and tissue) has provided us with a greater ability to focus on the gene expression changes that appear to be due to or in response to methapyrilene-induced liver injury.

## Materials and Methods

### Animals and treatment

Animal handling and treatment have been described in detail previously (Hamadeh et al. 2002). In brief, we dosed male Sprague-Dawley (Charles River Laboratories, Kingston, NY) rats for one, three, or seven daily doses by gavage with vehicle (water), 10 mg/kg/day methapyrilene, or 100 mg/kg/day methapyrilene (*n* = 4 rats per dose group). Twenty-four hours after the last dose, livers and kidneys were collected from animals euthanized by CO_2_ asphyxiation. Cross-sections of the left liver lobe and the left kidney were collected in 10% neutral-buffered formalin for histopathologic evaluation. The remaining left liver lobe and left kidney were minced and snap frozen in liquid nitrogen within several minutes after euthanasia for RNA isolation. Animals were treated humanely and with regard for the alleviation of suffering in accordance with the guidelines established in the Public Health Service Policy on Humane Care and Use of Laboratory Animals (National Institutes of Health 2002).

### Histopathology

Representative sections of liver and kidney from all animals were processed, embedded in paraffin, sectioned in 5-μm slices, and stained with hematoxylin and eosin. Microscopic histopathologic evaluations of the tissue sections were conducted by a veterinary pathologist and reviewed by a team of veterinary pathologists. Results from histopathologic analysis of the liver sections were reported in an earlier article (Hamadeh et al. 2002).

### RNA isolation

Isolation of liver RNA has been described previously (Hamadeh et al. 2002). We isolated and quantitated total RNA from kidney using the same protocol. For both liver and kidney samples, we pooled equal amounts of RNA from each of the four animals per treatment or control group for gene expression analysis.

### Microarray analysis

We prepared cRNA targets from 500 ng total RNA by following the protocol outlined in the Agilent Low RNA Input Fluorescent Linear Amplification Kit (Agilent Technologies, Santa Clara, CA). The cRNA targets from each treatment group’s pooled sample were hybridized to the Agilent Rat Oligo Microarray (G4130A, 22,575 probes representing more than 20,000 genes and expressed sequence tags; Agilent Technologies) in quadruplicate, with fluor reversals, against the time-matched pooled control sample according to Agilent’s Oligonucleotide Microarray Hybridization Protocol (Agilent Technologies 2007). Fluorescent intensities were measured with the Agilent Scanner and analyzed using Array Suite Software (version 7.1; Agilent Technologies). The resulting image and data files were loaded into Rosetta Resolver (version 5.1; Rosetta Biosoftware, Seattle, WA) for further analysis. Data from each treatment group’s four individual chips were combined in Resolver to achieve average fold changes and *p*-values according to Resolver’s Error Model (Weng et al. 2006). Differentially expressed genes identified by Resolver’s Error Model for each group (based on the four combined chips) were those with a *p*-value < 0.001, fluorescent intensity for at least one channel > 500, and absolute fold change > 1.2. In addition, we used an analytical program developed at the National Institute of Environmental Health Sciences (NIEHS), EPIG (extracting gene expression patterns and identifying co-expressed genes), to identify patterns of gene expression that differed between the liver and kidney (Zhou et al. 2006). EPIG identified genes belonging to a pattern according to the following criteria: signal-to-noise ratio > 3, correlation > 0.85, with a minimum of seven genes required to make up a pattern.

The microarray data generated for this study have been deposited in the National Center for Biotechnology Information’s Gene Expression Omnibus (GEO, http://www.ncbi.nlm.nih.gov/geo/; Edgar et al. 2002) and are accessible through GEO Series accession number GSE5381.

## Results

### Histopathology

A detailed histopathologic analysis of the rat livers from methapyrilene administration has been described previously (Hamadeh et al. 2002). Briefly summarized, high-dose methapyrilene (100 mg/kg) elicited hepatocellular necrosis, periportal lipid vacuolization, and biliary hyperplasia that increased in incidence and severity from one to seven doses. Low-dose methapyrilene (10 mg/kg) administration resulted in minimal hepatocyte necrosis after only seven doses. See Hamadeh et al. (2002) for representative images of liver histology after methapyrilene administration. No treatment-related observations were noted in the kidney after methapyrilene administration [see Figure 1 in Supplemental Material for representative images of kidney histology (http://www.ehponline.org/docs/2007/9396/suppl.pdf)], justifying the use of the kidney as a nontarget tissue for toxicity.

### Differential gene expression

To compare differential gene expression in the liver and kidney after methapyrilene treatment, RNA from livers and kidneys were hybridized to the Agilent rat oligonucleotide microarray chip. The liver RNA, which was previously analyzed with an in-house cDNA chip made at the NIEHS as previously reported by Hamadeh et al. (2002), was reanalyzed on the Agilent rat oligonucleotide chip for two reasons. First, using the same platform to analyze gene expression changes in the liver and the kidney facilitates the comparison of the two tissues (Weis and Consortium 2005). Second, the Agilent oligonucleotide chips contain greater content and have better annotation than our previous cDNA chips.

We used the Rosetta Resolver Error Model (Weng et al. 2006) to identify differentially expressed genes in each tissue at each time point for the two different doses of methapyrilene (Table 1). High-dose (100 mg/kg/day) methapyrilene administration resulted in the differential expression of thousands of genes in the liver at each time point, increasing from 1,736 genes after one dose to 3,123 genes after seven daily doses. A total of 4,258 genes in the liver were identified as being differentially expressed by high-dose methapyrilene administration for at least one time point, with over 700 genes differentially expressed at each time point. Of those genes found to be differentially expressed in the liver at each time point, more than 95% were either up-regulated or down-regulated at all three time points. Low-dose (10 mg/kg/day) methapyrilene administration resulted in the differential expression of fewer genes in the liver, with the greatest number of differentially expressed genes observed after only one dose (715 genes). Although a total of 1,245 genes were differentially expressed in the liver after low-dose methapyrilene administration for at least one time point, only 2 genes were consistently differentially expressed (down-regulated) across all three time points.

Compared with the differential gene expression observed in the liver, methapyrilene administration resulted in only 200 genes being differentially expressed in the kidney, ranging from 129 genes after three low doses to 267 after one high dose (Table 1). A total of 544 genes were identified as being differentially expressed in at least one time point in kidneys from animals treated with high-dose methapyrilene, whereas only 385 genes were identified as being differentially expressed in at least one time point in kidneys from animals treated with low-dose methapyrilene. Similar to what was observed in livers from low-dose animals, very few genes were consistently differentially expressed across all time points for each dose group, with only 8 and 2 genes consistently altered in high-dose– or low-dose–treated animals, respectively.

### Liver and kidney gene expression comparison

To identify gene changes specifically related to the hepatotoxicity of methapyrilene, we compared gene expression alterations in the liver with those in the kidney, under the assumption that genes with similar expression patterns in both the liver and kidney are not likely to be related to the toxic actions of methapyrilene. We make this assumption because no treatment-related pathologic lesions were observed in the kidney after methapyrilene administration. Because methapyrilene is bio-activated to toxic metabolites via cytochromes P450 in the liver (Ratra et al. 1998a) and undergoes extensive enterohepatic circulation (Ratra et al. 2000), we reasoned that biologically significant gene expression alterations would be of smaller magnitude in the kidney than in the liver. This was the case with smaller numbers of differentially expressed genes detected in the kidney compared with the liver (Table 1). Accordingly, using strict fold-change or *p*-value cutoffs might not have the required sensitivity to detect important gene changes in the nontarget tissue. However, because gene expression data were gathered from multiple time points after methapyrilene administration, it was possible to use the gene expression profiles over time to identify genes regulated in common and differentially in the two tissues. Thus, by examining the temporal profiles that genes display in each tissue, we can ascribe gene expression alterations to specific effects, such as those related to toxicity or the pharmacologic actions of methapyrilene.

**Figure 1:**
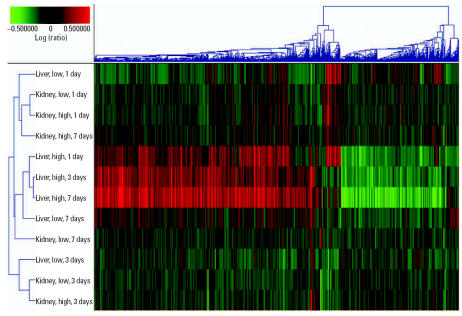
Hierarchical clustering of tissue samples after methapyrilene administration. Agglomerative hierarchical cluster (cosine correlation with average linkage) and heat map of liver and kidney samples after methapyrilene administration for one to seven doses across the 1,962 genes identified as being differentially expressed by EPIG. The liver samples cluster together primarily on the basis of the severity of toxicity elicited by methapyrilene, whereas the kidney samples cluster primarily on the basis of the number of doses received, regardless of dose level. Each sample listed contains the average gene expression value for four replicates. The heat map displays the average gene expression ratio of treated samples compared with time-matched control. Red indicates genes that are up-regulated after methapyrilene treatment; green indicates genes that are down-regulated after methapyrilene treatment; and black indicates no change in gene expression between methapyrilene-treated animals and vehicle-treated animals.

**Figure 2:**
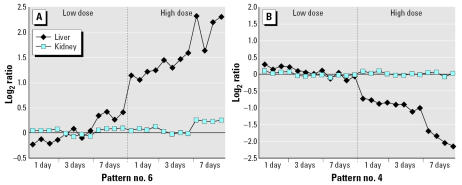
Gene expression patterns correlated to methapyrilene-mediated hepatotoxicity. EPIG identified patterns that contain genes with expression values that correlate with methapyrilene-induced hepatotoxicity. (*A*) EPIG pattern no. 6 contains 956 genes whose expression ratios increase with the number of high methapyrilene doses in the liver but no changes in the kidney. (*B*) EPIG pattern no. 4 contains 487 genes for which expression ratios decrease with the number of high methapyrilene doses in the liver but no changes in the kidney. Each point on the graph represents the average expression value of the top 5 genes in the pattern for each microarray performed. See Zhou et al. (2006) for a more detailed description of EPIG.

**Figure 3:**
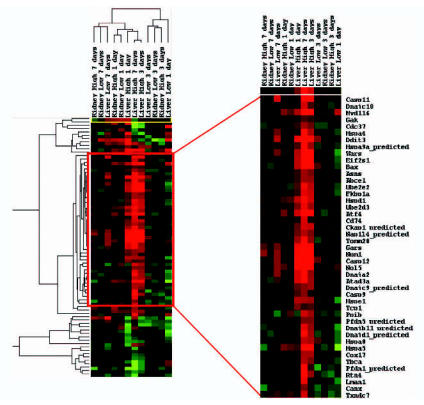
Hierarchical clustering of genes involved in ER stress and the unfolded protein response. Methapyrilene elicited up-regulation of numerous genes associated with ER stress and the unfolded protein response primarily in the high-dose liver only. Many of these genes are key regulators of ER stress, such as *Hspa5* and *Eif2s1*, whereas others, such as *Atf4* and *Gadd34/Myd116,* are transcripts downstream of the unfolded protein response. Using Gene Ontology (http://www.geneontology.org/GO.annotation.shtml) annotation for ER stress or unfolded protein binding, 75 genes were identified that were differentially expressed in high-dose liver in at least one time point. Table 1 in Supplemental Material (http://www.ehponline.org/docs/2007/9396/suppl.pdf) lists all 75 genes and their relative fold change after methapyrilene administration.

We used EPIG to examine the temporal profiles of gene expression in the liver and kidney after methapyrilene administration. Using the parameters described in “Material and Methods,” EPIG identified 17 distinct profiles of gene expression elicited by methapyrilene administration, which contained a total of 1,962 genes [see Supplemental Material for all EPIG patterns (http://www.ehponline.org/docs/2007/9396/suppl.pdf)]. Agglomerative, hierarchical clustering (cosine correlation with average linkage) was performed on all samples using the 1,962 genes selected by EPIG (Figure 1). The high-dose liver samples clustered together primarily because of the greatest changes in differential expression observed in these treatment groups. The low-dose liver sample at 7 days also clusters with the high-dose liver samples, which is consistent with the onset of hepatic damage observed after seven doses of 10 mg/kg methapyrilene (Hamadeh et al. 2002). The remaining low-dose liver samples and the kidney samples tend to cluster based upon the number of doses the animals received. From this clustering it is apparent that the liver samples cluster primarily on the basis of the degree of hepatotoxicity elicited by methapyrilene (i.e., a dose-dependent response), whereas the kidney samples cluster on the number of doses administered, regardless of dose. The greatest number of genes selected by EPIG belonged to two different profiles. One of these profiles (pattern no. 6) consisted of 956 genes up-regulated by methapyrilene in a dose- and time-dependent manner in the liver only, with no change in expression levels in the kidney (Figure 2A). Genes that were identified in pattern no. 6 are hypothesized to be involved in methapyrilene toxicity, either in eliciting toxicity or in response to toxicity, as the pattern mirrors very nicely the incidence and severity of histopathologic lesions observed in the liver (Hamadeh et al. 2002). Genes in this pattern are highly enriched for protein synthesis (53 genes encoding ribosomal proteins and 10 genes involved with the E48S initiation complex) and cell death/apoptosis (19 genes), which are indicative of the cell stress response, repair response, and structural remodeling that have been identified previously (Hamadeh et al. 2002; Waring et al. 2004). The other profile (pattern no. 4) was just the opposite: 487 genes down-regulated in a dose- and time-dependent manner in the liver only, again with no change in the kidney (Figure 2B). Genes in this pattern are highly enriched for cytochromes P450 (23 genes), lipid metabolism (38 genes), steroid metabolism (13 genes), and fatty acid β-oxidation (5 genes). As with the genes in pattern no. 6, these biological pathways and processes were identified in previous reports analyzing gene expression changes after methapyrilene exposure (Hamadeh et al. 2002; Waring et al. 2004). In addition to confirming earlier results, our analysis of gene expression changes after methapyrilene administration revealed that methapyrilene induces an endoplasmic reticulum (ER) stress response in the liver. Twenty-nine genes involved in the ER stress response and/or unfolded protein binding were identified in the hepatotoxicity patterns (nos. 1, 4, 6), including genes such as eukaryotic translation initiation factor 2, subunit 1 alpha (*Eif2s1*), heat shock 70 kDa protein 5 (*Hspa5*), and activating transcription factor 4 (*Atf4*). Extending these results to include all differentially expressed genes, regardless of EPIG pattern, we found that methapyrilene elicits a dose-dependent increase in the number and fold change of differentially expressed genes involved in the ER stress response and/or unfolded protein binding in the liver only, from 16 differentially expressed genes after seven low doses to 42 differentially expressed genes after one high dose up to 64 differentially expressed genes after seven high doses of methapyrilene [Figure 3; Table 1 in Supplemental Material (http://www.ehponline.org/docs/2007/9396/suppl.pdf)].

**Figure 4:**
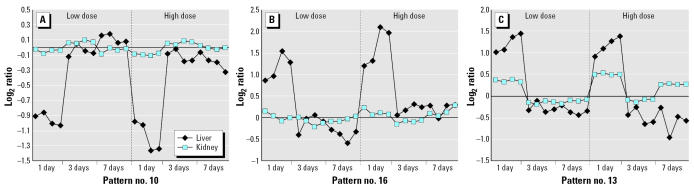
Gene expression patterns indicative of an acute response to methapyrilene administration. EPIG identified several expression patterns where the greatest change in gene expression was observed after a single dose. (*A*) EPIG pattern no. 10 (20 genes) with a dose-independent down-regulation of genes after a single dose of methapyrilene in the liver only. (*B*) EPIG pattern no. 16 (25 genes) with a dose-independent up-regulation of genes after a single dose of methapyrilene in the liver only. (*C*) EPIG pattern no. 13 (45 genes) with a dose-independent up-regulation of genes after a single dose of methapyrilene. Genes in pattern no. 13 exhibit similar changes in liver and kidney but with a higher magnitude of change in the liver.

Although many of the gene expression alterations identified by EPIG confirmed those that were previously published, the real strength of this analysis is the identification of those genes and processes that may be related to events other than methapyrilene-mediated hepatotoxicity. EPIG identified gene changes associated with a single administration of methapyrilene that were dose independent (pattern nos. 10, 13, and 16). The 89 genes in these three patterns (Figure 4) include genes involved in the acute stress response, such as heat shock protein 70 and α-2 macroglobulin. Because these genes are induced or repressed only after a single administration, regardless of dose, they appear to indicate a stress-related response due to methapyrilene administration. EPIG also identified genes with similar expression profiles in both the liver and kidney, which we consider less likely to be involved in hepatotoxicity. However, it is possible that adaptive mechanisms are present in the kidney that prevent toxicity [see EPIG pattern nos. 7, 8, 14, and 17 in Supplemental Material (http://www.ehpon-line.org/docs/2007/9396/suppl.pdf)]. The genes in these four separate patterns (a total of 264 genes) are involved in processes such as glucose homeostasis, protein transport and localization, and transcriptional regulation. Finally, EPIG identified genes specifically differentially expressed in the kidney rather than the liver. Pattern no. 9 [Supplemental Material (http://www.ehponline.org/docs/2007/9396/suppl.pdf)] contains genes that are up-regulated in the kidney after methapyrilene administration but down-regulated slightly in the liver, including several metallothionein genes and multidrug resistance protein 3.

## Discussion

The main goal of many toxicogenomics experiments is to use global gene expression profiling to gain insight into the mechanism underlying the organ damage elicited by the studied toxicant or environmental stressor. As most toxicogenomics experiments produce hundreds to thousands of gene expression changes resulting from toxicant exposure, trying to identify those gene changes that correspond to the cellular events involved in toxicity is quite daunting. Furthermore, the biological meaning underlying the observed gene expression changes is confounded by the multitude of tissue responses that occur with exposure to environmental agents, including but not limited to pharmacologic responses, nonspecific acute stress responses, toxic responses, adaptive responses, and recovery responses. Proper study design can help reduce the complexity of biological interpretation of the results. Many toxicogenomics studies incorporate multiple doses and multiple time points into the study design (Amin et al. 2004; Bulera et al. 2001; Hamadeh et al. 2004, 2002; Heijne et al. 2003; Heinloth et al. 2004; Kier et al. 2004; Lühe et al. 2003; Ruepp et al. 2002), but based on results from this study, we also recommend obtaining gene expression data from a nontarget tissue, in addition to using multiple dose levels and time points, for the purpose of clarifying the biological relevance of the observed expression changes in the target tissue for toxicity.

We chose to use the kidney as the nontarget tissue for methapyrilene hepatotoxicity primarily because methapyrilene administration did not induce any pathologic lesions in the kidney. Once an appropriate nontarget tissue had been selected, the question that remained was how to best use the information gained from analyzing gene expression changes in the nontarget tissue. Not surprisingly, methapyrilene administration resulted in relatively few gene expression changes at each time point in the kidney compared with the liver (Table 1) when using fold-change and *p*-value criteria to establish significance. However, examining differential gene expression at each time point in isolation does not take advantage of the study design employed in this experiment, namely, two different doses and three time periods of methapyrilene exposure.

We used the analytical program EPIG to identify significant changes in gene expression on the basis of their pattern of expression over time, including both doses and the two tissues. EPIG identified biologically significant genes by using each gene’s signal magnitude and signal-to-noise ratio to categorize them into patterns of expression in an unsupervised manner (Zhou et al. 2006; Chou JW, Zhou T, Paules RS, Kaufman WK, Bushel PR, unpublished data), based on the assumption that genes within the same pattern are co-expressed because they have a regulator in common or they are involved in the cellular events that the pattern describes. The advantages of using the temporal patterns of gene expression to identify biologically relevant genes are severalfold. First, this method of analyzing gene expression will help exclude false positives by including only genes in patterns that have similar profiles to other genes. Second, it is more straightforward to correlate patterns of gene expression with the observed histologic changes that occur over time. Third, it is highly likely that biologically relevant genes that are changed because of a perturbed cellular process will have similar profiles of expression. Fourth, for this experiment it is not necessary to have strict fold-change or *p*-value cutoffs, so that a wider range of gene changes can be identified. This is particularly useful in this experiment because of the different sensitivities of the two tissues to methapyrilene. The liver is highly sensitive to methapyrilene-mediated toxicity, in part because of the considerable enterohepatic circulation of methapyrilene and its metabolites (Ratra et al. 2000). Consequently, the kidney is not exposed to the same levels of methapyrilene as the liver. In addition, the oral route of administration will also ensure that the liver is exposed to higher levels of methapyrilene than the kidney because of the first-pass effect.

The results provided by EPIG analysis of the gene expression patterns confirms the gene changes identified in the previously published reports (Hamadeh et al. 2002; Waring et al. 2004). However, our analysis differs in that we have identified select groups of genes that we can reasonably ascribe to different physiologic or pathologic processes that occur because of methapyrilene administration. The greatest number of gene expression alterations occurs in two patterns (nos. 4 and 6), which show changes in expression in a dose- and tissue-specific manner; significant changes occurred in the liver but not in the kidney. This is especially encouraging in that it implies that the major biological event, namely, hepatotoxicity, is responsible for the majority of the observed gene changes, as these two patterns correlate well with the development of hepatotoxicity elicited by methapyrilene. Our analysis confirms that methapyrilene-mediated hepatotoxicity is accompanied by induction of genes involved in protein translation, structural remodeling, cell death/apoptosis, and the repression of several metabolic processes including lipid metabolism, steroid metabolism, and fatty acid β-oxidation.

In addition to the repression of genes involved in cellular metabolism, high-dose methapyrilene administration resulted in the down-regulation of numerous cytochrome P450 enzymes, as previously reported (Hamadeh et al. 2002; Waring et al. 2004; Wrighton et al. 1991). Our analysis indicates that the concomitant down-regulation of the nuclear receptors that regulate cellular and xenobiotic metabolism–related gene expression may be the underlying cause for the observed repressed pathways. Farnesoid X receptor [*FXR/Nr1h4*; GenBank accession no. NM_021745 (http://www.ncbi.nlm.nih.gov/Genbank/)] and pregnane X receptor (*PXR*/*Nr1i2*; GenBank accession no. NM_052980), which have been shown to maintain cholesterol and lipid homeostasis (Guo et al. 2003; Lambert et al. 2003), are down-regulated in high-dose livers only (pattern nos. 4 and 1, respectively). Coupled with the down-regulation of *PXR*, methapyrilene also induced a liver-dependent down-regulation of the constitutive androstane receptor (*CAR/Nr1i3*; GenBank accession no. NM_022941) and retinoid X receptor alpha (*RXRa*; GenBank accession no. NM_012805), possibly accounting for the repression of numerous xenobiotic-metabolizing enzymes (Beigneux et al. 2002; Wei et al. 2002). Besides their roles in differentially regulating the expression of genes involved in various metabolic functions, the repression of these receptors/transcription factors may explain, at least in part, the hepatotoxic actions of methapyrilene. The down-regulation of *PXR* may contribute to the hepatotoxicity of methapyrilene, as the ligand-induced activation of PXR serves to protect against bile acid toxicity resulting from several hepatotoxicants (Staudinger et al. 2001), whereas the resistance to bile acid toxicity is lost in mice lacking *PXR* (Xie et al. 2000). Additionally, PXR and CAR work in concert to protect against cholestatic injury induced by bile duct ligation (Stedman et al. 2005). The down-regulation of *PXR* and *CAR*, along with *FXR* (Guo et al. 2003), would make the liver more susceptible to bile acid toxicity, which, coupled with the biliary hyperplasia, may exacerbate the hepatotoxic effects of methapyrilene administration.

Our gene expression analysis also suggests another novel aspect underlying methapyrilene-mediated hepatotoxicity, namely, ER stress resulting from the unfolded protein response [reviewed by Boyce and Yuan (2006)]. ER stress and the unfolded protein response have been shown to be involved in hepatotoxicity resulting from acetaminophen (Macanas-Pirard et al. 2005), cyclohexamide (Ito et al. 2006), ethanol (Ji and Kaplowitz 2003), and hepatitis C virus (Benali-Furet et al. 2005). The unfolded protein response is activated by the influx of unfolded proteins into the ER lumen that exceed the processing capacity of the ER to restore ER homeostasis [reviewed by Patil and Walter (2001); Schroder and Kaufman (2005)]. Several key chaperones are up-regulated only in the liver after methapyrilene administration, including heat shock 70 kDa proteins 5 and 9a and several genes in the DnaJ (Hsp40) homolog family [Figure 4; Table 1 in Supplemental Material (http://www.ehponline.org/docs/2007/9396/suppl.pdf)]. Furthermore, ER stress-inducible genes, such as *Atf4*, *Gadd34/Myd116,* and *Ddit3/CHOP,* are up-regulated in the liver after high-dose methapyrilene administration. Even though the unfolded protein response is activated to restore ER homeostasis, if the stress is too strong or prolonged, ER stress can lead to cellular apoptosis [reviewed by Boyce and Yuan (2006); Patil and Walter (2001); Schroder and Kaufman (2005)]. Our results also suggest that the ER stress elicited by methapyrilene leads to hepatocellular apoptosis/cell death, as evidenced by the up-regulation of genes involved in apoptosis/cell death, including caspase 9, caspase 11, caspase 12, and BCL-associated X protein. The exact mechanism through which methapyrilene induces ER stress and the unfolded protein response is currently unknown, but several possibilities exist. Oxidative stress, which may induce or be a consequence of ER stress (Cullinan and Diehl 2006), has been reported to be associated with methapyrilene hepatotoxicity (Ratra et al. 1998b), although other reports suggest that oxidative stress is not involved in methapyrilene-mediated hepatotoxicity (Ratra et al. 2000). Additionally, the unfolded protein response may be activated by the methapyrilene-mediated perturbations in cellular lipid metabolism, as excess saturated fatty acids have been shown to alter ER homeostasis in liver cells, resulting in ER stress and apoptosis (Wei et al. 2006). It is also possible that biliary damage associated with methapyrilene hepatotoxicity induces ER stress, as bile acids have been shown to elicit this stress (Bernstein et al. 1999; Payne et al. 2005).

By examining the expression profiles in both the liver and kidney (at various doses and times), we have also been able to identify genes, biological processes, and pathways unlikely to be involved in methapyrilene hepatotoxicity. EPIG identified three profiles that are indicative of an acute response to methapyrilene administration, that is, either induction or repression after a single dose of methapyrilene only. One might consider these genes to be involved in hepatotoxicity; however, all three of the profiles indicate that the gene expression alterations are independent of dose in the liver, and one profile shows similar changes in the kidney as well. Thus, these genes and pathways can be ruled out in our analysis of hepatotoxicity, as they likely represent a generalized acute stress response to methapyrilene administration. For example, several genes involved in glucose homeostasis are altered after a single dose in both the liver and kidney. This implies that methapyrilene administration transiently alters glucose handling and production; however, the tissues are able to resume normal glucose homeostasis by the third dose of methapyrilene.

Our analysis of the gene expression data has also changed the interpretation of some of the processes that have previously been reported to be involved in methapyrilene-mediated hepatotoxicity. Methapyrilene was reported to elicit oxidative stress in the liver, as evidenced by the up-regulation of genes involved in glutathione production and other genes involved in the response to oxidative stress (Waring et al. 2004). However, most of these genes were not selected by EPIG as belonging to any of the identified patterns, possibly because the selection of these genes is differentially expressed at a single time point in the previous analysis. If that were the case, then they would not be identified in EPIG as being biologically relevant, under the presupposition that biologically relevant gene expression alterations show concordant patterns with other important genes. As an example, many glutathione *S*-transferases are up-regulated in the liver after only seven high doses of methapyrilene, but only glutathione *S*-transferase a5 was identified by EPIG in a toxicity-related pattern (pattern no. 6). Rather than being directly related methapyrilene-mediated hepatotoxicity, the altered expression of genes involved in glutathione production and oxidative stress may be secondary to the accumulation of reactive biliary metabolites (Ratra et al. 2000). In addition, Waring et al. (2004) observed alterations in genes involved in the sterol/retinol pathway; however, EPIG did not identify these genes as belonging to a pattern, again suggesting that these gene alterations are not directly related to hepatotoxicity.

In conclusion, our data show that comparing gene expression changes between target and nontarget tissues for toxicity can help clarify the biological interpretation of identified gene changes. Additionally, examining gene changes across multiple time points and multiple doses is more advantageous than comparing gene changes at each time point in isolation, which has the potential of missing biologically relevant genes. By examining gene expression across tissue, dose level, and number of doses, we were able to filter out genes that are in common between target and nontarget tissue, as these reflect nonspecific properties of the compound administration. Furthermore, gene changes that appear to be due to a nonspecific stress response can also be filtered out, thus leaving a more focused set of genes to concentrate on in our understanding of the cellular events that occur during and after compound-mediated toxicity.

## Figures and Tables

**Table 1:** Number of differentially expressed genes in liver and kidney after methapyrilene administration.

Group	Genes	Up-regulated	Down-regulated	Union	Up intersection	Down intersection
Liver						
100 mg/kg, 1 day	1,736	1,074	662			
100 mg/kg, 3 days	2,216	1,504	712	4,258	554	191
100 mg/kg, 7 days	3,123	2,153	970			
10 mg/kg, 1 day	715	333	382			
10 mg/kg, 3 days	349	77	272	1,245	0	2
10 mg/kg, 7 days	414	229	185			
Kidney						
100 mg/kg, 1 day	267	170	97			
100 mg/kg, 3 days	183	54	129	544	2	6
100 mg/kg, 7 days	226	125	101			
10 mg/kg, 1 day	202	133	69			
10 mg/kg, 3 days	129	29	100	385	1	1
10 mg/kg, 7 days	143	71	72			

Animals were treated with one, three, or seven daily doses of methapyrilene (10 or 100 mg/kg/day). Differential gene expression in liver and kidney were measured 24 hr after the last dose using the Agilent rat chip. Differentially expressed genes were identified using the Rosetta Resolver Error Model using the following criteria: intensity > 500 in at least one channel, absolute fold change > 1.2, and *p* < 0.001. The second column lists the total number of genes differentially expressed in the given treatment group; the third and fourth columns list the number of genes up-regulated and down-regulated, respectively, in each treatment group. The “Union” column lists the union of all the differentially expressed genes across all three treatment periods for each tissue and dose group. The last two columns list the intersection of the up-regulated and down-regulated genes, respectively, across all three treatment periods for each tissue and dose group.
